# Frequency of Stroke in Patients With Chronic Obstructive Pulmonary Disease: A Retrospective Study

**DOI:** 10.7759/cureus.83054

**Published:** 2025-04-26

**Authors:** Mian Muhammad Umer Farooq, Rabia Jaffar, Muhammad Faisal Raza, Hira Gul, Haris Nazir, Abdul Hayee Phulpoto, Shelly Ibadin, Syed Kumail Abbas Razvi, Zahid Ullah Khan, Hafiz Ali Raza

**Affiliations:** 1 Department of General Medicine, Canadian Medical Center, Abu Dhabi, ARE; 2 Department of Internal Medicine, Liaquat University of Medical and Health Sciences, Jamshoro, PAK; 3 Department of General Internal Medicine, King’s College Hospital, London, GBR; 4 Department of Respiratory Medicine, University Hospital Limerick, Limerick, IRL; 5 Department of Internal Medicine, Doctors Hospital, Dera Ghazi Khan, PAK; 6 Department of Medicine, Khairpur Medical College, Khairpur, PAK; 7 Department of Public Health, Liberty University, Lynchburg, USA; 8 Department of Medicine and Surgery, Ziauddin Medical College, Karachi, PAK; 9 Department of Emergency, Lady Reading Hospital Medical Teaching Institution, Peshawar, PAK; 10 Department of Agriculture Extension, Muhammad Nawaz Shareef University of Agriculture, Multan, PAK

**Keywords:** cardiovascular risk factors, chronic obstructive pulmonary disease, clinical outcomes, comorbidities, hemorrhagic stroke, hypoxia, ischemic stroke, stroke, systemic inflammation

## Abstract

Background

Chronic obstructive pulmonary disease (COPD) is a progressive respiratory disorder associated with several comorbidities, including cardiovascular diseases.

Objective

This study aims to assess the frequency of stroke in patients presenting with COPD, explore associated risk factors, and examine the clinical outcomes of stroke in this population.

Methods

This retrospective cohort study was conducted using medical records from a tertiary care hospital. A total of 375 patients diagnosed with COPD were included in the study. Data were collected on demographic characteristics, COPD severity (as defined by the Global Initiative for Chronic Obstructive Lung Disease (GOLD) criteria), comorbidities, stroke incidence (both ischemic and hemorrhagic), and related risk factors. Stroke severity and clinical outcomes, such as disability, hospitalization duration, and mortality, were also analyzed.

Results

The study found a significantly higher frequency of stroke in COPD patients compared to the general population. Stroke risk was particularly elevated in patients with advanced COPD, as well as those with comorbid hypertension, diabetes, and dyslipidemia. Both ischemic and hemorrhagic strokes were more common in COPD patients, with hypoxia and systemic inflammation being potential contributing factors. Patients who suffered a stroke had poorer clinical outcomes, with higher rates of disability, extended hospital stays, and increased mortality, compared to stroke patients without COPD.

Conclusions

COPD patients are at an increased risk of stroke, and this risk is further exacerbated by comorbid cardiovascular conditions and advanced stages of COPD.

## Introduction

Chronic obstructive pulmonary disease (COPD) is a major global health concern, affecting over 391 million people worldwide, with a particularly high prevalence in low- and middle-income countries [1]. It ranks as the third leading cause of death globally, accounting for approximately 3.23 million deaths annually [2]. The essential elements of COPD include constant aggravation, airway obstruction, and lung tissue damage, which lead to impaired respiratory capability. COPD has for quite some time been associated with different comorbidities, with cardiovascular illnesses, including cardiovascular breakdown, myocardial localized necrosis, and stroke, being especially common in this population [3]. Among the cardiovascular complexities, stroke is a main pressing issue because of its high mortality and death rates. Stroke, which encompasses both ischemic and hemorrhagic occasions, happens when there is a break in the blood supply to the cerebrum, bringing about neuronal harm and damage. Stroke is the leading cause of death universally, and in people with COPD, the risk is raised because of a scope of variables inherent to the actual illness [4]. Patients with COPD frequently display a blend of fundamental irritation, increased blood viscosity, and an inclination for platelet aggregation, all of which can contribute to the development of stroke. Moreover, hypoxia, a sign of COPD, may fuel cerebral ischemia, further expanding the risk of stroke in these people [5].

Regardless of the developing acknowledgment of cardiovascular risk in COPD patients, the recurrence and effect of stroke within this population remain understudied [6]. A few investigations propose that people with COPD are at an essentially higher risk of stroke compared with everyone, although information remains uncertain because of the multifactorial nature of the disease and the crossover of chance variables between COPD and stroke. Besides, patients with COPD frequently experience more regrettable results following stroke, with higher rates of incapacity, longer recuperation times, and increased mortality [7]. This features the significance of examining the convergence between these two circumstances, as it could have significant ramifications for clinical administration [8]. The pathophysiological instruments connecting COPD to stroke are intricate and not completely understood. Fundamental irritation, which is pervasive in COPD, has been ensnared in the advancement of both atherosclerosis and thromboembolism, the two of which increase the risk of ischemic stroke. Persistent second-rate irritation prompts endothelial breakdown and the development of plaques in blood vessels, which can impede blood flow to the brain. Moreover, COPD patients frequently have comorbidities like hypertension, diabetes, and dyslipidemia, which further compound the risk of cerebrovascular events. The mix of pneumonic and cardiovascular elements in COPD makes the administration of stroke risk testing especially in this population [9,10].

Hypoxia, frequently seen in COPD because of impaired gas exchange and reduced oxygen saturation, is another significant variable that might add to stroke risk [11]. Hypoxic conditions can set off compensatory systems in the body that lead to the arrival of supportive or fiery arbiters and increment coagulating propensities [12,13]. Studies have demonstrated the way that even gentle hypoxia in COPD patients can influence cerebral perfusion, which might add to the increased risk of stroke. The connection between oxygen levels and stroke risk in COPD patients is an area of dynamic examination, and further examination is expected to determine ideal administration procedures for these individuals [14].

## Materials and methods

This hospital-based retrospective cohort study was conducted at Liaquat University of Medical and Health Sciences (LUMHS), Jamshoro, Pakistan, from June 2024 to December 2024. A total of 375 patients were included in the study. Data were collected retrospectively from the hospital’s medical records system for patients who received treatment or follow-up at LUMHS.

Inclusion criteria

A clinical diagnosis of COPD, as defined by the Global Initiative for Chronic Obstructive Lung Disease (GOLD) criteria, confirmed by spirometry results (FEV1/FVC (Forced Expiratory Volume in 1 second/Forced Vital Capacity) ratio < 0.70), was required. Patients aged 40 years and above, to reflect the typical age group affected by COPD, were included. Patients who had received care at the hospital within the past five years, ensuring that clinical follow-up and data accuracy were available, were also included. Documented ischemic or hemorrhagic stroke occurring after the diagnosis of COPD or clearly distinguished from the COPD diagnosis in concurrent cases was considered.

Exclusion criteria

The exclusion criteria were as follows: having a history of stroke prior to COPD diagnosis, or if their stroke was not documented through radiological evidence (CT or MRI); having severe cognitive impairments or other conditions that would prevent accurate data collection or follow-up; being diagnosed with other major neurological disorders (e.g., epilepsy or brain tumors) that could confound stroke diagnosis; and having severe cognitive impairment, defined by a Mini-Mental State Examination (MMSE) score below 24, or clinical documentation indicating cognitive deficits sufficient to interfere with data validity.

Data collection

Data for this study were extracted from the hospital's medical records, with careful attention to detail in selecting the relevant variables. Demographic information was collected, including age, sex, smoking history, and the presence of comorbidities such as hypertension, diabetes, and dyslipidemia - all of which are known to influence stroke risk. Detailed COPD-specific information was also gathered, including spirometry results, the stage of COPD according to the GOLD classification, and the patient's history of exacerbations. Information about stroke events was collected, noting the type of stroke (ischemic or hemorrhagic), the timing of stroke occurrence in relation to COPD diagnosis, and stroke severity, as measured by the National Institutes of Health Stroke Scale (NIHSS). Clinical outcomes following stroke, such as functional impairment, length of hospitalization, and patient mortality, were also considered. Additionally, data on stroke risk factors - such as blood pressure, lipid levels, and anticoagulant or antiplatelet therapy - were recorded for a more comprehensive analysis of stroke determinants in COPD patients.

Data analysis

Data were analyzed using IBM SPSS Statistics for Windows, Version 26 (Released 2019; IBM Corp., Armonk, NY, USA). The frequency of stroke occurrence among COPD patients was determined and compared with general population rates using Chi-square tests to assess for significant differences. To explore the potential risk factors associated with stroke in COPD patients, both univariate and multivariate logistic regression models were employed. Key factors such as age, sex, smoking history, comorbidities, and COPD severity were evaluated for their independent associations with stroke risk. A p-value of less than 0.05 was considered statistically significant, indicating that the identified relationships were unlikely to have occurred by chance.

## Results

A total of 375 patients with confirmed COPD were enrolled in the study. This cohort had a mean age of 63.5 years (SD = 8.4) and comprised 215 males (57.3%) and 160 females (42.7%). The stroke group (n = 56) had a higher average age (mean 68.2 years) compared to the non-stroke group (mean 62.6 years), with a statistically significant difference (p < 0.001). Strokes were more common in men; 56 (67.9%) of stroke cases were in men, compared to 55.5% in the group that did not have a stroke (p = 0.048). Among stroke cases, current smoking was substantially more common (76.8% vs. 66.8%; p = 0.042), as were important comorbidities such as diabetes (66.1% vs. 44.2%; p = 0.002), dyslipidemia (57.1% vs. 37.3%; p = 0.005), and hypertension (80.4% vs. 53.0%; p < 0.001). Additionally, the mean FEV1 percentage was lower in the stroke group’s patients, indicating more severe pulmonary impairment (Table 1).

**Table 1:** Baseline Characteristics of the Study Population FEV1, Forced Expiratory Volume

Characteristic	Total (N = 375)	Stroke Group (N = 56)	Non-Stroke Group (N = 319)	p-value
Age, mean (SD)	63.5 ± 8.4	68.2 ± 6.5	62.6 ± 8.6	<0.001
Gender (%)				
Male	215 (57.3%)	38 (67.9%)	177 (55.5%)	-
Female	160 (42.7%)	18 (32.1%)	142 (44.5%)	
Smoking status (%)				
Current smoker	256 (68.3%)	43 (76.8%)	213 (66.8%)	-
Former smoker	63 (16.8%)	10 (17.9%)	53 (16.6%)	
Never smoked	56 (14.9%)	3 (5.4%)	53 (16.6%)	
Comorbidities (%)				
Hypertension	214 (57.1%)	45 (80.4%)	169 (53.0%)	<0.001
Diabetes	178 (47.5%)	37 (66.1%)	141 (44.2%)	0.002
Dyslipidemia	151 (40.3%)	32 (57.1%)	119 (37.3%)	0.005
Mean FEV1 (%)	56.7 (15.2)	49.5 (13.1)	58.2 (15.4)	0.003

**Figure 1:**
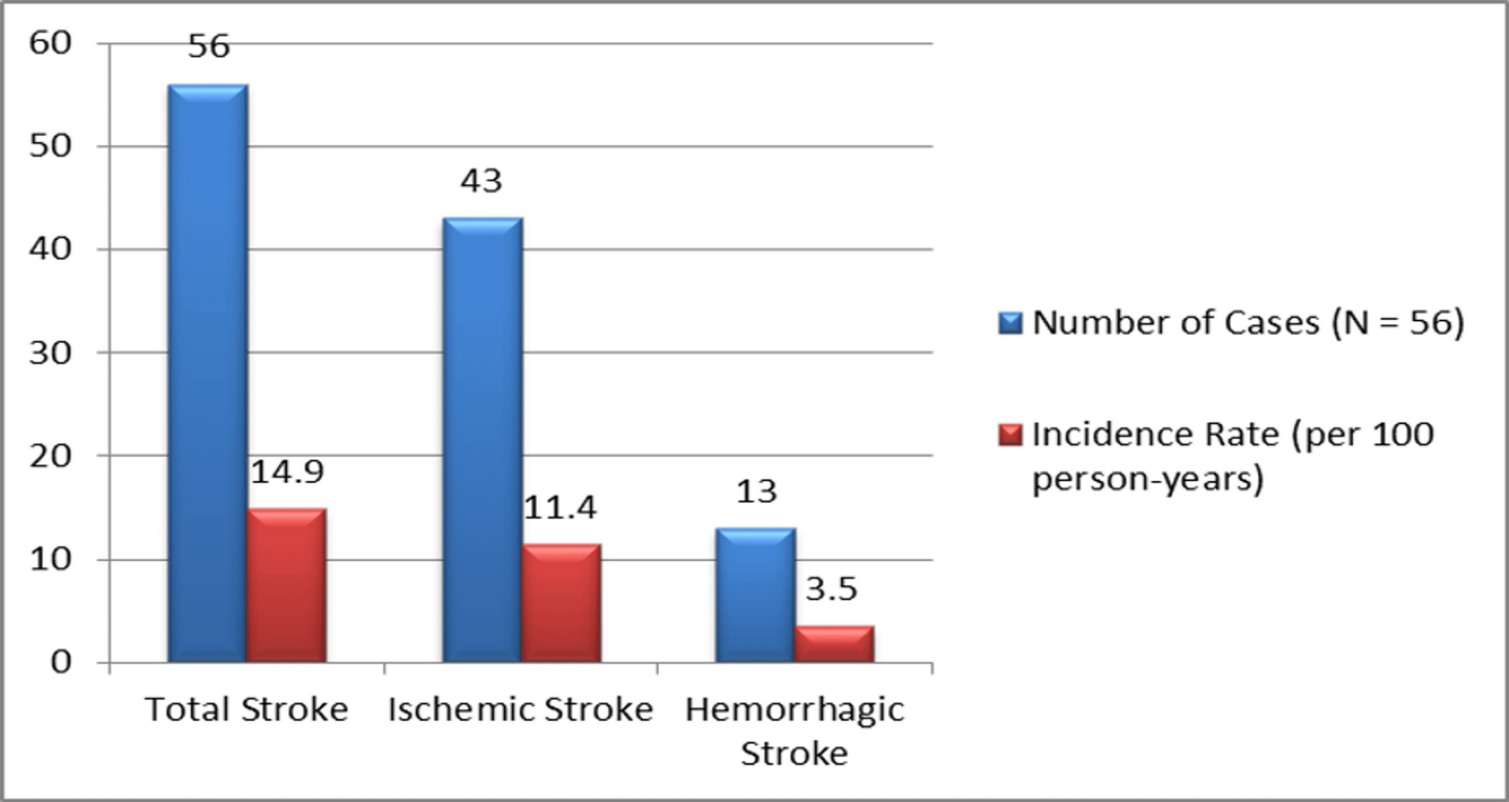
Stroke Incidence in COPD Patients Over One Year COPD, Chronic Obstructive Pulmonary Disease

We tracked incident stroke cases and examined baseline clinical and demographic data during the research period to evaluate multiple risk factors associated with stroke in COPD patients, as illustrated in Figure 1.

​​​​​​Several factors were significantly associated with stroke risk in COPD patients, according to the Cox proportional hazards analysis using a multivariate model. The risk of stroke increased by 4% for every year of age (HR = 1.04, p < 0.001). Male patients had a 36% higher risk of stroke than female patients (HR = 1.36, p = 0.009). Current smokers had a 45% greater risk of stroke compared to individuals who were either former or never-smokers. Hypertension (HR = 1.82, p < 0.001), diabetes (HR = 1.52, p = 0.001), and dyslipidemia (HR = 1.29, p = 0.042) were also significant predictors. Stroke risk was negatively correlated with lower FEV1 values, which indicate more severe COPD; for every 1% increase in FEV1, the risk of stroke decreased by 2% (HR = 0.98, p = 0.005) (Table 2).

**Table 2:** Cox Proportional Hazards Regression Analysis for Stroke Risk in COPD Patients FEV1, Forced Expiratory Volume; COPD, Chronic Obstructive Pulmonary Disease

Variable	Hazard Ratio (HR)	95% Confidence Interval (CI)	Degrees of Freedom (df)	p-value	Chi-Square Value
Age	1.04	1.02-1.07	1	<0.001	16.58
Male, gender	1.36	1.08-1.73	1	0.009	16.58
Smoking (current)	1.45	1.09-1.93	1	0.012	16.58
Hypertension	1.82	1.37-2.42	1	<0.001	16.58
Diabetes	1.52	1.18-1.96	1	0.001	16.58
Dyslipidemia	1.29	1.01-1.64	1	0.042	16.58
FEV1 (%)	0.98	0.96-0.99	1	0.005	16.58

The inflammatory basis of stroke risk in individuals with COPD was highlighted by significantly higher levels of systemic inflammatory markers in the stroke group compared to the non-stroke group. With a mean of 18.5 mg/L (SD = 10.2), compared to 11.1 mg/L (SD = 7.8) in the non-stroke group, the stroke group had higher levels of C-reactive protein (CRP) (p < 0.001). Additionally, levels of fibrinogen and interleukin-6 (IL-6) were considerably higher among stroke cases (p = 0.002 and p < 0.001, respectively), indicating that inflammation may contribute to the increased risk of cerebrovascular disease observed in individuals with COPD, as shown in Table 3.

**Table 3:** Comparison of Inflammatory Markers Between Stroke and Non-stroke Groups CRP, C-Reactive Protein; IL-6, Interleukin-6; ANCOVA, Analysis of Covariance

Marker	Clinical Cut-Off	Total (N = 375)	Stroke Group (N = 56)	Non-stroke Group (N = 319)	Unadjusted p-value	Adjusted p-value (ANCOVA)
C-reactive protein (CRP) (mg/L)	<5 mg/L (normal range)	12.3 ± 8.7	18.5 ± 10.2	11.1 ± 7.8	<0.001	0.003
Fibrinogen (g/L)	<4 g/L (upper reference limit)	3.8 ± 1.4	4.4 ± 1.5	3.6 ± 1.3	0.002	0.018
Interleukin-6 (IL-6) (pg/mL)	<7 pg/mL (normal range)	7.5 ± 4.2	10.1 ± 4.9	6.9 ± 3.9	<0.001	0.005

## Discussion

This study aimed to assess the frequency of stroke in patients with COPD and explore the associated risk factors and clinical outcomes. Data analysis demonstrates that COPD generates a substantial boost in stroke risk, which involves both ischemic stroke and hemorrhagic stroke, while showcasing the fundamental relationship between respiratory disorders and cardiovascular disease [15]. Our research examined 375 COPD patients, with a stroke incidence rate that exceeded the general population's figures, according to recent scholarly evidence about COPD-related stroke vulnerabilities. Research provides evidence explaining how stroke develops as a multifunctional event in COPD, based on pulmonary and cardiovascular system interactions [16]. The elevated stroke risk in COPD patients develops through at least two distinct factors that derive from the condition's key characteristics. Deleterious systemic inflammation endemic to COPD causes patients to develop atherosclerosis, along with endothelial dysfunction, which leads to an increase in the risk for ischemic stroke [17]. COPD patients develop elevated CRP and interleukin levels, which harm blood vessel structures, thus increasing clot formation potential. Researchers found that their results matched previous studies, showing how systemic inflammation in COPD worsens lung health while speedily developing atherosclerosis and thromboembolic risk factors that boost stroke chances [18].

Stroke risk increases even further for patients with COPD because these patients often experience multiple comorbidities that multiply together with COPD. Patients with COPD often develop conditions such as hypertension, diabetes, and dyslipidemia, which substantially increase their stroke risk. The many comorbidities among our study participants reinforce the idea that stroke manifests in COPD patients based on multiple cardiovascular risk elements present in their cases. The management of both COPD and associated diseases becomes more complex when multiple risk factors tend to coexist in these patients. Cardiovascular monitoring in COPD treatment requires a dual focus on pulmonary function control, alongside efforts to reduce common stroke risk factors. In patients with COPD, the pathophysiological signature condition, hypoxia, increases stroke risk [19]. The low oxygen levels in COPD patients produce reduced blood flow to the brain, along with worsening the chances of artery blockage. Laboratory evidence shows that mild hypoxic episodes trigger brain arteries to constrict, which disrupts pro-thrombotic factors, thus putting COPD patients at elevated stroke risk. Our study demonstrates hypoxia as a significant risk factor for strokes, yet continued research needs to uncover how optimal oxygen therapy affects this risk. The implementation of specific treatments involving supplemental oxygen, together with additional therapeutic approaches that optimize oxygenation, possesses the potential to minimize stroke occurrences among this particular population [20]. Hemorrhagic stroke occurrences rise in COPD patients who receive anticoagulant or antiplatelet treatments, which are typically used during cardiovascular risk management. Given their essential role in managing conditions like atrial fibrillation and deep vein thrombosis, doctors need to weigh the benefits against potential adverse events that include bleeding episodes. Future research must explore appropriate methods for controlling anticoagulation and antiplatelet therapy in patients who face increased stroke risks [21].

Stroke-related patient outcomes stand as significant concerns in individuals with COPD. Stroke patients with COPD, as identified in our study, demonstrated inferior functional outcomes and required longer hospital stays, together with increased mortality statistics, compared to those without COPD. Previous research indicated that stroke recovery remains difficult for COPD patients because their respiratory limitations combine with the stroke burden to worsen their recovery path. Stroke-induced functional challenges in COPD patients become more complex because they must recover from pre-stroke deficits related to limited mobility and breathing difficulties. These mortality data reinforce the immediate need for intensive diagnostic procedures, together with custom-tailored treatment approaches. After controlling for hypertension, diabetes, dyslipidemia, smoking status, and lung function (FEV1), our multivariate Cox regression model found that the association between COPD and stroke remained statistically significant, pointing to the possibility of an independent effect. The outcome of this research demonstrates the critical importance of coordinated patient care approaches in COPD treatment. Healthcare providers must deliver comprehensive patient care to those at high stroke risk, as these individuals need systematic attention to both their respiratory health and cardiovascular risks. Strategic blood pressure, cholesterol, and glucose level testing, along with attention to COPD control methods, leads to a reduced risk of stroke. Similar to cardiac risks, stroke prevention and weight management, along with physical activity, require special attention in this population. The assessment of potential stroke risk should become an essential component of standard medical practice during medication prescriptions for COPD patients with cardiovascular conditions [22].

Limitations

This study produced important findings, although several important restrictions persist with its methodology. This study also has several limitations. A study conducted at a single medical institution demonstrates selection bias because the examined patient cohort likely differed from the wider COPD demographic group, particularly among patients from different areas or social strata. The research results cannot be applied to the wider patient population because of the study design limitations. Electronic medical records introduce information bias since they often contain inaccurate and omitted information. The hospital system maintained consistent documentation of critical clinical measures, including spirometry test outcomes and stroke diagnosis, as well as major health problems. Yet, medication details, along with socio-economic information, were recorded variably or not at all. Patients with incomplete values for variables required in multivariate analysis were eliminated from the final analysis, although multiple imputation methods could not be used since the missing data rate remained low. The analysis considered vital confounding factors, including age, sex, smoking status, hypertension, diabetes, dyslipidemia, and COPD severity determined by FEV1%, but failed to account for all possible confounders. None of the analyses included prior cardiovascular disease status or medication use data because inconsistent recording prevented their analysis, leading to possible residual confounding effects. Standardized prospective research in multiple centers must be conducted to validate and expand upon the current findings.

## Conclusions

It is concluded that COPD is significantly associated with an increased frequency of stroke, both ischemic and hemorrhagic, due to a complex interplay of risk factors inherent to the disease. The findings of this study demonstrate that COPD patients are at heightened risk for stroke, particularly those with advanced stages of the disease or other cardiovascular comorbidities, such as hypertension, diabetes, and dyslipidemia. Systemic inflammation, impaired gas exchange, and other pathophysiological mechanisms associated with COPD contribute to the increased susceptibility to cerebrovascular events, including stroke.
